# Acute aortic syndromes after esophageal endoscopic submucosal dissection

**DOI:** 10.1186/s12877-025-06664-4

**Published:** 2025-11-29

**Authors:** Jing Pang, Erfeng Li, Wenbin Zhang, Feng Wang, Bin Guo, Jiwei Ren

**Affiliations:** Shanxi Province Cancer Hospital/Shanxi Hospital Affiliated to Cancer Hospital, Chinese Academy of Medical Sciences/Cancer Hospital Affiliated to Shanxi Medical University, Taiyuan, 030001 China

**Keywords:** Early esophageal cancer, Endoscopic submucosal dissection (ESD), Acute aortic syndromes (AAS)

## Abstract

**Background:**

Endoscopic Submucosal Dissection (ESD) is established as a primary therapeutic approach for early esophageal cancer, with perforation, bleeding, and stricture recognized as the most significant complications. Current evidence regarding acute aortic syndromes (AAS) following ESD remains limited.

**Case presentation:**

A previously healthy, 66-year-old woman presented with half a month history of early esophageal cancer through upper gastrointestinal endoscopy. Upper gastrointestinal endoscopy examination showed: Approximately 21–25 cm away from the incisors, a type II-b lesion on the left posterior wall of the esophagus. Eight days post-hospitalization, she underwent esophageal ESD. Seven hours post-operation, she experienced sudden chest pain. An urgent chest CT scan suggested aortic intramural hematoma(IMH). Following transfer to the vascular surgery department for symptomatic supportive treatment, her condition stabilized, leading to her discharge. Follow-up at three and six months confirmed the aortic condition remained stable. The occurrence of AAS post-ESD is exceedingly rare, with minimal reports in the published literature.

**Conclusions:**

This study reviews the major complications associated with ESD and explores the potential link between esophageal ESD and AAS.

**Supplementary Information:**

The online version contains supplementary material available at 10.1186/s12877-025-06664-4.

## Introduction

Endoscopic Submucosal Dissection (ESD) is recognized as the mainstay treatment for early esophageal cancer, with perforation, bleeding, and stricture acknowledged as major complications. Reports of AAS following ESD are notably scarce, suggesting that AAS is an exceptionally rare complication of ESD. Herein, we detail a case of aortic intramural hematoma post-esophageal ESD.

## Case presentation

A previously healthy 66-year-old woman with no history of tobacco, alcohol, or illicit drug use presented with early esophageal cancer detected by upper gastrointestinal endoscopy. There was no family history of gastrointestinal tumors. Physical examination and laboratory tests, including complete blood count, liver and kidney function tests, and tumor markers (CEA, CA199, CA724, CA125, AFP), were unremarkable. An electrocardiogram showed normal findings.

**Fig. 1:**
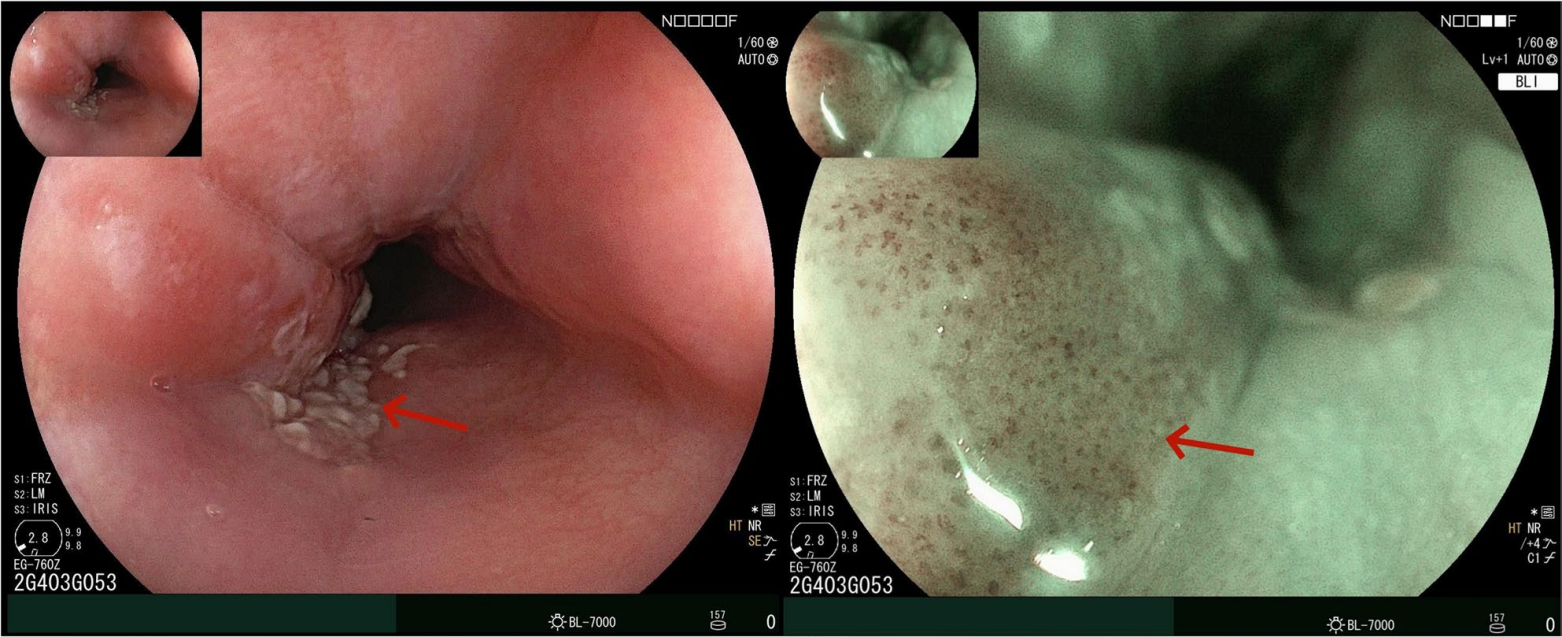
Upper gastrointestinal endoscopy examination. *Upper gastrointestinal endoscopy revealed a solitary type II-b lesion at 21–25 cm from the incisors, characterized by surface roughness. Under BLI electronic chromoendoscopy, the lesion exhibited brownish discoloration. Magnification endoscopy demonstrated atypical microvessels. **The arrow indicates the the lesion site

Upper gastrointestinal endoscopy revealed a type II-b lesion on the left posterior wall of the esophagus, located 21–25 cm from the incisors, demonstrating surface roughness (Fig. 1). Chest and abdominal CT showed no esophageal wall thickening. Biopsy results indicated high-grade intraepithelial neoplasia of squamous epithelium. She underwent esophageal ESD on the eighth day of hospitalization.

**Fig. 2:**
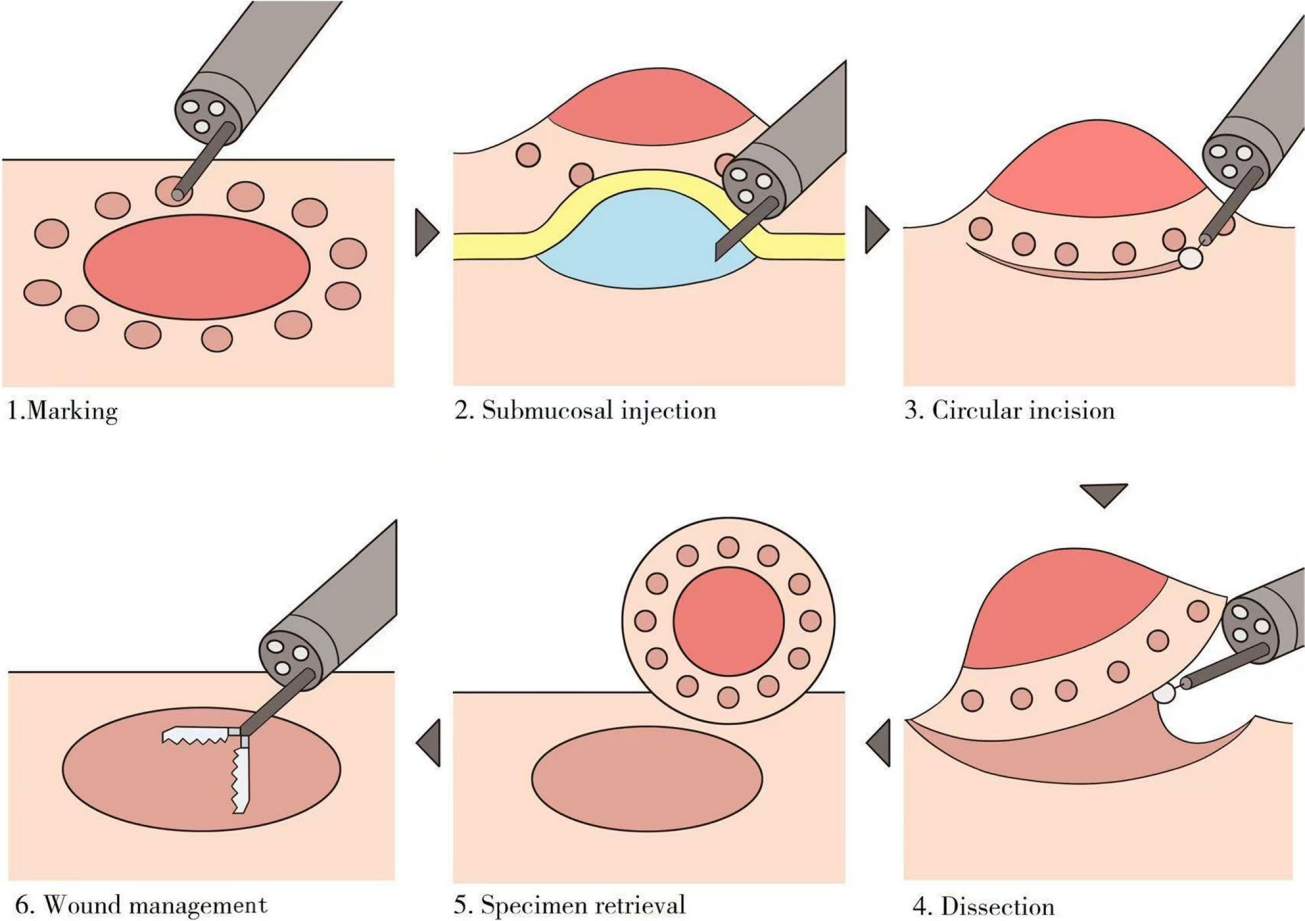
Key Procedural Steps of ESD. *This schematic illustrates the standardized key steps of esophageal endoscopic submucosal dissection (ESD): 1.Marking: Circumferential electrocautery demarcation is performed 2–3 mm beyond the lesion margins. 2.Submucosal injection: A viscous solution is injected to create a sustained submucosal fluid cushion, separating the mucosal layer from the muscularis propria. 3.Circular incision: A full-thickness mucosal incision is made along the marked points to establish the dissection plane. 4.Dissection: Submucosal layer-by-layer dissection is performed under direct visualization. 5.Specimen retrieval: The resected specimen is carefully retrieved and pinned for pathological evaluation of lateral/vertical margins. 6.Wound management: Final hemostasis and verification of muscularis propria integrity

**Fig. 3:**
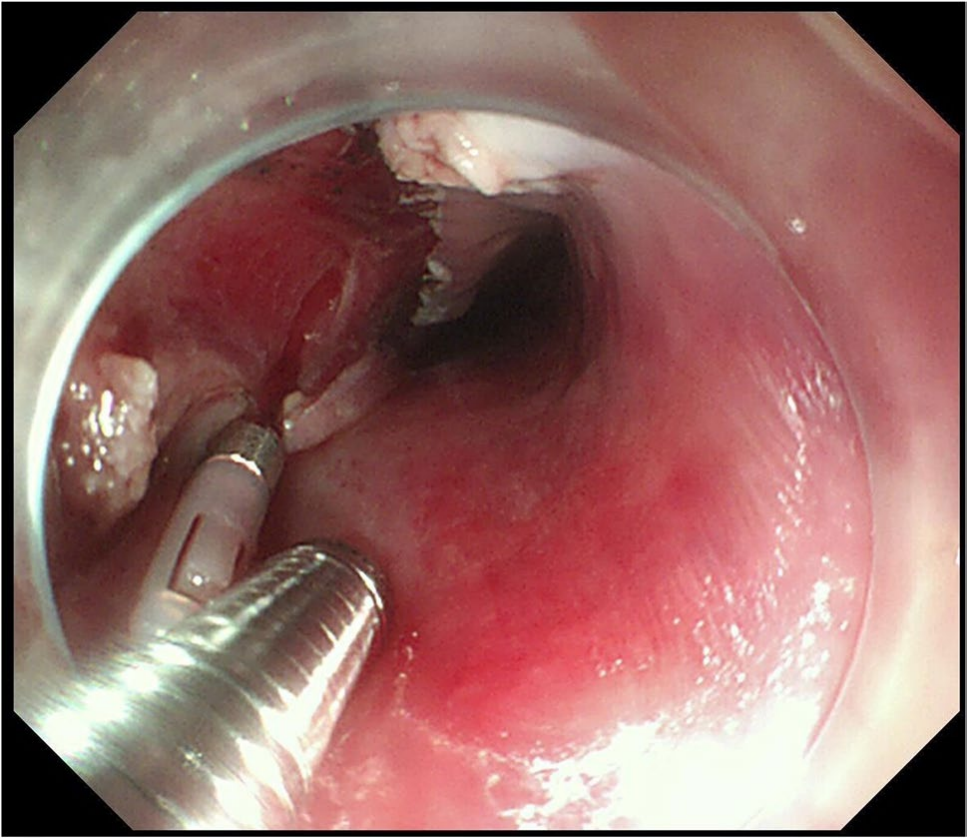
Endoscopic images immediately after ESD. *During coagulation at the resection bed, focal deep thermal injury occurred at the right lesion margin, exposing the muscularis propria (circular layer). No perforation was observed, and prophylactic placement of one titanium clip was performed

Pre-anesthetic medication includes intravenous dexamethasone 5 mg and atropine 0.5 mg. Anesthesia induction commenced at 11:35 with sequential IV boluses of etomidate 20 mg, sufentanil 20 µg, and cisatracurium 20 mg, followed by successful endotracheal intubation at 11:49. Maintenance was achieved via continuous infusions of remifentanil (30 ml/h of 0.004% solution), dexmedetomidine (0.5 µg/kg/h), and propofol (3.3 µg/ml/h). The surgical incision occurred at 12:00. At 12:12, intraoperative monitoring revealed hypotension (85/40 mmHg), which was attributed to hypovolemia. Hemodynamics stabilized after an IV dopamine 2 mg bolus and fluid resuscitation. Subsequently, blood pressure was maintained at 110–120/60–70 mmHg, heart rate at 70–80 bpm, and oxygen saturation at 96% (spontaneous) and 99% (mechanical). ECG showed sinus rhythm, ETCO₂ ranged from 36 to 38 mmHg, and Bispectral Index (BIS) values decreased sequentially from 94 (awake state) to a plateau of 48–52 (surgical anesthesia depth), then increased to 85 (emergence consciousness). Mucosal dissection was performed using an Olympus DualKnife KD-650 L and Coagrasper FD-410LR with ERBE VIO 200 S generator settings (Cut: 3-2-4; Forced coagulation: 2–45 W). The surgical procedure is illustrated in Fig. 2. Physiological saline methylene blue solution (100 mL + 0.5 mL) was used for successive submucosal injections, with a total dosage of approximately 50 ml. During coagulation at the resection bed, focal deep thermal injury exposing the muscularis propria (circular layer) occurred at the right lesion margin (Fig. 3). Despite muscular layer exposure, no perforation was observed, prompting prophylactic placement of one titanium clip. The procedure was completed with minimal intraoperative hemorrhage. Hemostasis was achieved using forced coagulation. The operation concluded successfully at 13:00. Extubation was performed at 13:10. The patient was transferred to the ward in stable condition at 13:24. Postoperative management included strict bed rest, NPO status, acid suppression, anti-infective prophylaxis, and symptomatic treatment with nutritional fluid replenishment. Blood pressure was maintained within 125–135/70–75 mmHg.

**Fig. 4:**
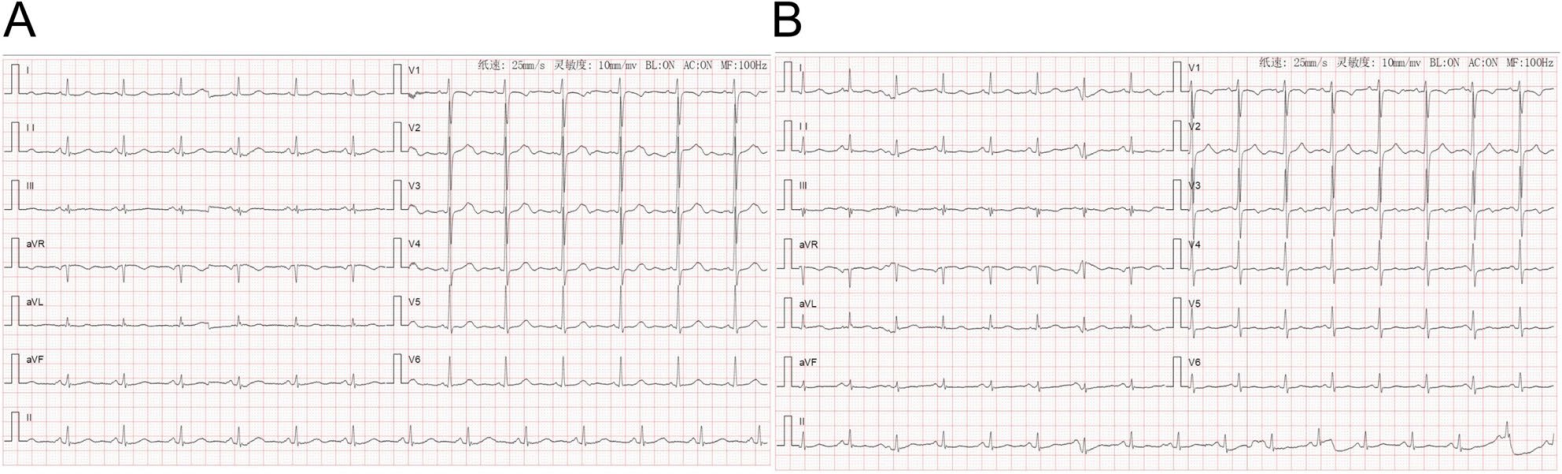
Electrocardiogram. *A Preoperative electrocardiogram showed sinus rhythm with essentially normal findings. **B** Postoperative electrocardiogram demonstrated sinus rhythm with T-wave changes

**Fig. 5:**
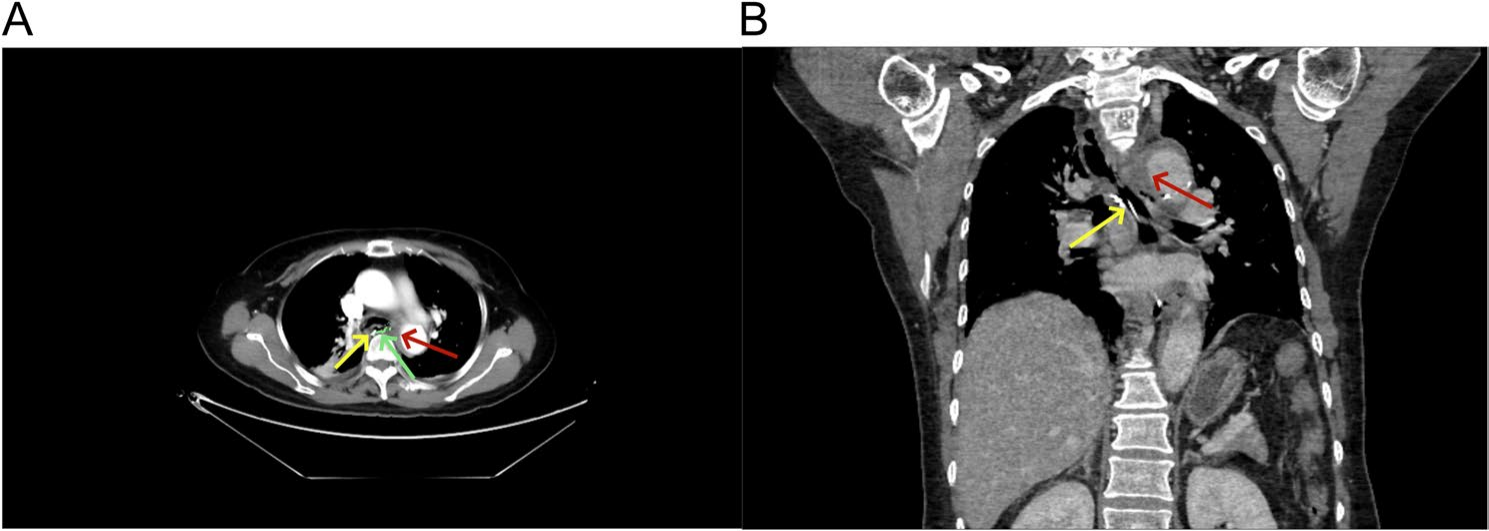
Postoperative Chest computed tomography. *A: Axial CT image; **B**: Coronal reconstructed CT image; **Yellow arrow denotes titanium clip position; red arrow indicates intramural hematoma; green arrow and line delineate the approximate lesion location

**Fig. 6:**
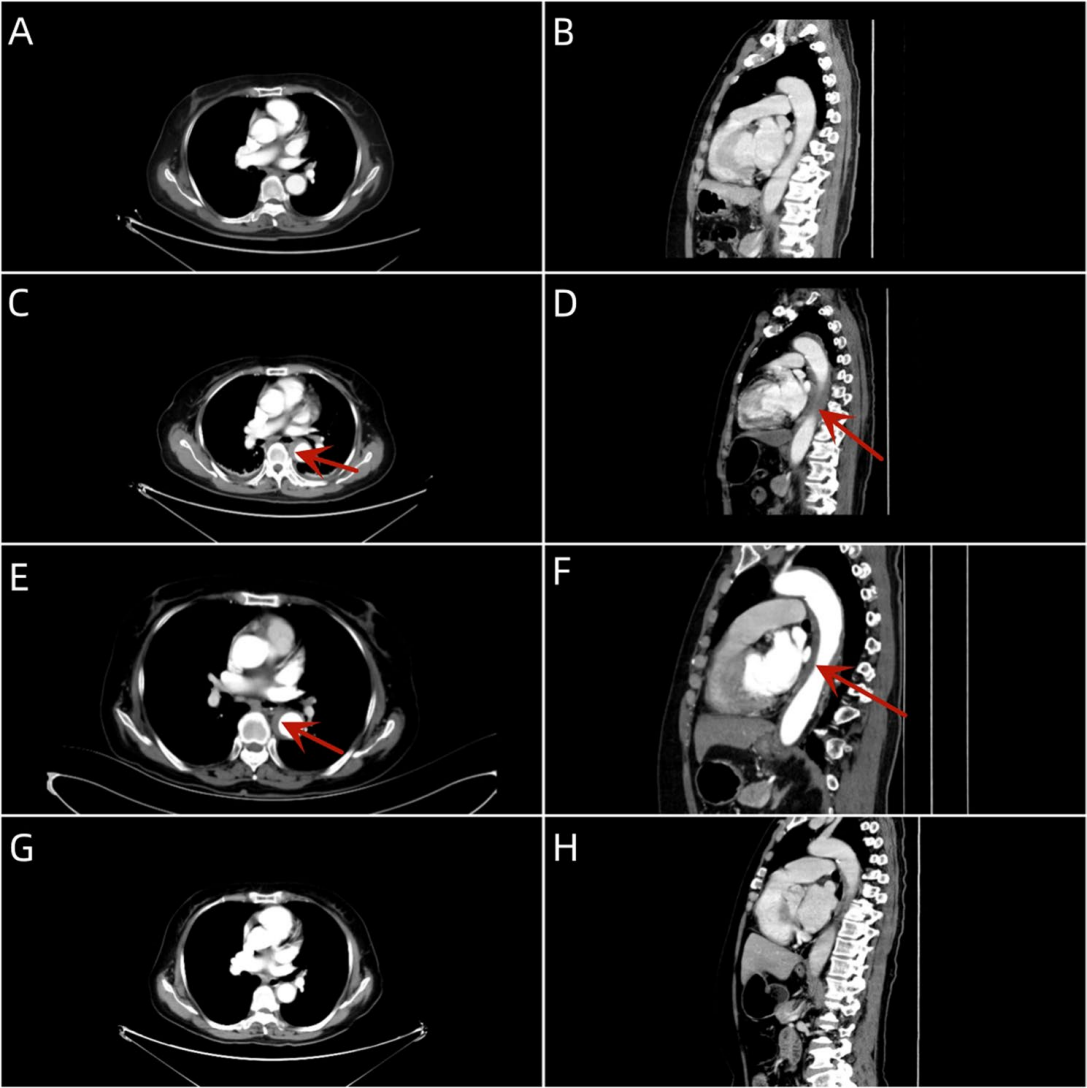
Chest computed tomography. *A: Preoperative axial CT image; **B**: Preoperative sagittal reconstructed CT image; **C**: Axial CT image at 7 h post-procedure; **D**: Sagittal reconstructed CT image at 7 h post-procedure; **E**: Axial MPR at postoperative day 7; **F**: Sagittal MPR image at postoperative day 7; **G**: Axial CT image at 10 months post-procedure; **H**: Sagittal reconstructed CT image at 10 months post-procedure; **Red arrow denotes intramural hematoma location

Seven hours post-operation, she developed sudden chest pain. Initial vital signs were: blood pressure 125/60 mmHg, heart rate 78 beats/min, and blood oxygen saturation 97%. Physical examination revealed no signs of peripheral ischemia, including absence of pain, pallor, paresthesia, paralysis, or coolness. Laboratory tests showed: Complete Blood Count with WBC 7.90 × 10⁹/L (Neutrophils 75.8% [5.99 × 10⁹/L]), RBC 4.15 × 10¹²/L, HGB 128 g/L, and PLT 218 × 10⁹/L. B-type Natriuretic Peptide was 110.00 pg/mL. Biochemistry results included Urea 6.0 mmol/L, Creatinine 60.0 µmol/L, CO₂ 22.8 mmol/L, Glucose 7.9 mmol/L, CK 75.1 IU/L, CK-MB 7.9 IU/L, LDH 266.7 IU/L, α-HBDH 163.7 IU/L, K⁺ 4.46 mmol/L, Na⁺ 128.9 mmol/L, Cl⁻ 98.2 mmol/L, Phosphate 0.66 mmol/L, Magnesium 0.72 mmol/L, Total Calcium 2.21 mmol/L, and Homocysteine 9.4 µmol/L. Cardiac markers included CK-MB mass 1.4 ng/mL, Myoglobin 26.5 ng/mL, and hs-TnI 2.60 pg/mL. The electrocardiogram exhibited T-wave changes(Figure 4). Chest CT (Fig. 5) revealed circumferential and crescent-shaped low-density shadows around the aortic arch to the level of the aorta piercing the diaphragm, with clear boundaries and a small amount of calcified plaque noted. No definitive intimal tear was observed, suggesting the possibility of aortic intramural hematoma༈IMH༉. After discussion, we opted for medical management with blood pressure control, pain management, and prompt transfer to the vascular surgery department. Subsequently, following aggressive supportive management, the patient achieved clinical stability and was discharged on postoperative day 8 (Fig. 6).

## Discussion

As detailed in the global report on cancer incidence and mortality, esophageal cancer accounted for 510,716 new cases and 445,129 fatalities in 2022 [1]. Advances in diagnostic capabilities have led to increased detection of early-stage esophageal cancer. ESD has emerged as the primary treatment for early esophageal cancer, owing to its high resection rates, low recurrence rates, precise pathological diagnoses, and improved patient survival. However, the anatomical intricacies of the esophagus render ESD more technically challenging and prone to complications, with an overall incidence as high as 3% despite advancements in surgical techniques and instruments [2]. The most frequent complication post-ESD is stenosis, occurring in 5% of cases. The incidence of post-ESD esophageal stenosis exceeds 90% when the postoperative mucosal defect involves more than 3/4 of the luminal circumference. The application of a polyglycolic acid (PGA) sheet, systemic corticosteroid administration and local steroid injections may effectively prevent post-procedural stenosis, while endoscopic balloon dilatation (EBD) and radial incision and cutting (RIC) are recognized as effective therapeutic approaches [3–6]. Acid suppressants are also recommended to enhance ulcer healing and reduce the risk of postoperative bleeding [7]. Perforation, a grave complication that can lead to mediastinal emphysema or mediastinitis, occurs at a rate of 3% and includes both intraoperative and delayed perforations. Traction-assisted ESD has been demonstrated to significantly reduce the risk of intraoperative perforation. Delayed perforations may result from tissue necrosis and degeneration in the muscularis propria, potentially caused by heat denaturation due to excessive use of high-frequency electric knives [8]. High-frequency electric knives can cause two types of burns: burns at the electrode plate, and non-polar plate burns that occur away from the electrode plate. In 2014, Hsiang-Ling Wu et al. described a case of a patient with isolated squamous cell carcinoma the right posterior wall of the lower esophagus who developed hypertension, tachycardia, and subcutaneous emphysema in the lower neck and anterior chest wall during ESD at the 45-minute mark. Endoscopic visualization revealed cavitary structures, and emergent CT confirmed acute Stanford type B aortic dissection [9]. Additionally, except for a single documented instance of an infected aneurysm in a patient with a history of hemodialysis postoperatively, no other vascular complications related to ESD have been reported [10].

AAS is characterized by acute, tearing chest pain that often radiates to the back. It encompasses a spectrum of severe, life-threatening conditions, including aortic dissection (AD), IMH, and penetrating atherosclerotic ulcer (PAU) [11, 12]. Traditional risk factors for AAS, such as age over 60 years, male sex, uncontrolled hypertension, atherosclerosis, and connective tissue disorders, are associated with the compromise or disruption of the aortic wall. The hemodynamic stress of arterial blood flow can induce intimal tears, allowing blood to enter the false lumen and precipitating AD. IMH, resulting from bleeding within the aortic media, is widely considered an integral part of the pathophysiological process of aortic dissection [13–16]. The literature documents cases of aortic dissection occurring during percutaneous coronary intervention, with cross-clamping injury sites, suture lines, and cannulation sites posited as potential origins for subsequent dissection. Furthermore, the role of a thin, fragile, and dilated aorta in the pathogenesis of AAS is considered significant [17, 18].

Following sudden-onset chest pain approximately 7 h post-procedure, esophageal perforation was initially suspected. Subsequent chest CT imaging, however, demonstrated IMH. Although the incidental nature of this post-ESD intramural hematoma cannot be excluded, its potential pathophysiological relationship with the procedure warrants closer scrutiny. We propose that the development of AAS following esophageal ESD may be associated with several factors. First, the patient was a 66-year-old female with preexisting mild aortic calcification. Second, the esophageal wall, composed solely of muscular layers lacking a serosal layer, is inherently thin and vulnerable. Situated in the posterior mediastinum, the esophagus is in close proximity to the aorta, vertebral body, and trachea, subjecting it to compression and the dynamic influences of cardiac and respiratory movements, as well as the internal ring muscle. The posterior esophageal wall lesion (at the T4 vertebral level) was anatomically constrained by the anterior margin of the vertebral body and aortic arch, limiting operative maneuverability. Third, the posterior esophageal wall lies in close proximity to the anterior wall of the descending aorta (mean distance: 4–8 mm). Within this surgical field, thermal transmission from high-frequency electrosurgical currents may potentially compromise vasa vasorum integrity via diffusion of inflammatory mediators. During ESD, the left lateral positioning of the patient may induce pressure or stretching on the aorta, which could theoretically elevate the risk of AAS. The potential pathological process is as follows: Pre-existing patient-specific risk factors and individual variations resulted in aortic wall thinning and fragility. During esophageal ESD, rupture of the vasa vasorum or hemorrhage within the aortic media may have contributed to the development of AAS. Furthermore, transient hypertension induced by postoperative pain during anesthesia emergence may exert shear stress on the aortic wall with pre-existing pathology, potentially disrupting its structural integrity and initiating the pathological cascade. In this instance, prompt detection and intervention averted more dire outcomes. However, the high-frequency electrosurgical incisions during ESD were non-hemorrhagic and non-full-thickness injuries (depth: 2–3 mm), characterized by narrow and flat profiles. A direct mechanical association between these lesions and AAS requires further investigation for definitive validation.

Although a direct causal link between AAS and ESD has not been confirmed, clinicians should carefully evaluate ESD candidates for relevant risk factors.High-risk patients warrant further assessment of vascular wall integrity. Maintaining intraoperative hemodynamic stability, performing precise operative techniques, and implementing postoperative analgesia and blood pressure control strategies are imperative to mitigate AAS risk. As ESD becomes increasingly indispensable for treating superficial esophageal carcinomas, it is crucial to recognize that despite its minimally invasive nature, this procedure is not devoid of potential complications. Consequently, close postoperative surveillance is essential for the early detection and management of these complications.

## Conclusions

The current investigation enumerates the predominant complications associated with ESD and deliberates on the potential correlation between esophageal ESD and AAS. A thorough investigation into the underlying mechanisms may equip clinical physicians with the knowledge to preemptively mitigate the risk of associated complications.

## Supplementary Information

Supplementary Material 1

## Data Availability

The datasets used and/or analysed during the current study are available from the corresponding author on reasonable request.

## Abbreviations

ESD: Endoscopic Submucosal Dissection
BIS: Bispectral Index
AAS: Acute Aortic Syndromes
NPO: Nil Per Os
PGA: Polyglycolic acid
EBD: Endoscopic Balloon Dilatation
RIC: Radial incision and cutting
AD: Aortic Dissection
IMH: Aortic Intramural Hematoma
PAU: Penetrating Atherosclerotic Ulcer
